# High-Intensity Interval Training (HIIT): Impact of Duration on Body Composition, Cardiometabolic Health, and Aerobic Capacity in Adolescent Women

**DOI:** 10.3390/metabo15090623

**Published:** 2025-09-19

**Authors:** Mima Stankovic, Ilma Čaprić, Luka Pezelj, Emir Biševac, Raid Mekić, Armin Zećirović, Zerina Salihagić, Aldina Ajdinović, Igor Jelaska

**Affiliations:** 1 Faculty of Sport and Physical Education, University of Niš, 18000 Niš, Serbia; 2 Department of Biomedical Sciences, State University of Novi Pazar, 36300 Novi Pazar, Serbia; icapric@np.ac.rs (I.Č.); ebisevac@np.ac.rs (E.B.); rmekic@np.ac.rs (R.M.); zsalihagic@np.ac.rs (Z.S.); aajdinovic@np.ac.rs (A.A.); 3 Faculty of Maritime Studies, University of Split, 21000 Split, Croatia; luka.pezelj@pfst.hr; 4 Faculty of Sports and Physical Education, University of East Sarajevo, 71000 Sarajevo, Bosnia and Herzegovina; armin.zecirovic@gmail.com; 5 Faculty of Kinesiology, University of Split, 21000 Split, Croatia

**Keywords:** interval-based exercise, lipid metabolism, cardiovascular fitness, metabolic risk factors, training

## Abstract

Background: High-intensity interval training (HIIT) is a time-efficient approach that has been recognized to enhance cardiometabolic health and aerobic capacity in adolescents. The purpose of this study was to investigate the effects of various HIIT durations on cardiometabolic health and aerobic ability in adolescent women aged 17 to 19 years. Methods: Participants were separated into two intervention groups: HIIT 1 (6 weeks) and HIIT 2 (8 weeks), along with a control group. Both HIIT regimens included two weekly sessions: warm-up (jogging, accelerated running, and dynamic stretching), major sets (2 × 6–9 bouts of 30 s training at 90–95% HR_max_ with active recovery), and cooldown. Pre- and post-intervention measurements included body mass, BMI, body fat percentage, lipid profile, blood pressure, fasting glucose, and VO_2max_. Results: Both HIIT programs resulted in significant reductions in body weight, BMI, and body fat percentage (all *p* < 0.001), as well as improvements in total cholesterol, triglycerides, LDL cholesterol, and systolic and diastolic blood pressure (all *p* < 0.001), compared to the control group. The changes in glycemia (*p* = 0.078) and HDL cholesterol (*p* = 0.825) were not statistically significant. Both HIIT groups showed significantly higher VO_2max_ (*p* < 0.001). Conclusions: Adolescent women’s cardiometabolic health and aerobic capacity increased considerably following 6- and 8-week HIIT training. These findings emphasize HIIT as a practical and time-saving strategy for this population, highlighting its effectiveness in improving key health parameters within a relatively short period.

## 1. Introduction

Adolescence is crucial for establishing and implementing beneficial behaviors [1]. During the mentioned period, there are great physical, cognitive, emotional, and social changes, and it is characterized by dynamic development [2]. Adolescents’ optimal health is strongly impacted by frequent regular exercise, which promotes appropriate physical and mental growth [3]. Furthermore, insufficient physical exercise has been related to an increased chance of getting cardiovascular disorders, therefore raising the possibility of premature death [4].

Although regular physical activity contributes to prevent a variety of health problems, evidence shows that over the past few years, adolescents often regularly fall short of attaining the recommended physical activity guidelines [5]. According to studies, young women are significantly less physically active compared to men [6]. Particularly, 24.2% of young women were deemed insufficiently active, in contrast to 17.6% of men [7]. Hormonal fluctuations during adolescence, especially in girls, significantly affect physical performance, body composition, and response to training. Estrogen and progesterone fluctuate during the menstrual cycle, which can affect muscle strength, endurance, and recovery. Understanding these factors is critical to designing effective training programs for teenage girls [8].

Physical activity provides multiple health benefits across various physiological systems and is widely recognized as an important approach to manage cardiometabolic health [9]. Furthermore, it is recommended that they participate in muscle- and bone-strengthening exercises a minimum of three times every week. Nevertheless, one of the most significant hurdles for consistent physical activity among youngsters today is lack of time [10]. As a result, the usefulness of different types of physical activity must be evaluated. High-intensity exercise regimens have been shown to be more efficient than low-intensity sessions in addressing time constraints [11]. High-intensity training (HIIT) is an effective strategy to enhance health in a short amount of time, as time constraints are a key barrier to regular exercise [12]. Additionally, it minimizes the risk of cardiovascular disease in healthy young people [13].

Previous research suggests that HIIT can have a positive effect on cardiometabolic health and aerobic capacity, often emphasizing its time efficiency and potentially greater degree of enjoyment compared to continuous moderate-intensity exercise [14]. However, findings are not always consistent, as certain HIIT protocols, such as high-intensity Tabata training, have been rated as less enjoyable than continuous protocols in untrained young adults [15]. To improve cardiorespiratory health and fitness, physical activity sessions should last a minimum of 7 to 12 weeks, according to research. In a study by Foster et al. [15] in young women of normal body weight, an 8-week HIIT led to significant improvements in aerobic capacity, despite a shorter duration. In addition, in a randomized study, Frimpong et al. [16], in young women with normal and overweight body mass, a 14-week home HIIT program (six times per week) led to improvements in cardiorespiratory fitness, body composition, and cardiometabolic markers (systolic/diastolic blood pressure, lipid profile, and glycemic parameters). Furthermore, a study conducted on healthy young women of normal body weight, both HIIT protocols (classical and multimodal) during a 12-week program led to significant increases in VO_2max_ and muscle mass, while decreasing body fat percentage. According to the research, Lu et al. [17], involving young female students, both HIIT-R and high-intensity functional training (HIFT) led to an improvement in aerobic capacity (VO_2max_: +17.1–12.7%), as well as a decrease in body fat (body fat%: −17.1–12.6%) after 12 weeks. Although it was previously thought that significant increases in cardiorespiratory fitness required protocols lasting more than 12 weeks, there is growing evidence that adaptations occur in shorter periods of time. HIIT can improve mitochondrial biogenesis, insulin sensitivity, and oxidative enzyme activity within less than 12 weeks, leading to a rapid increase in VO_2max_ and an improved metabolic profile [18]. These data show that shorter HIIT protocols may also be beneficial in initial adaptations, necessitating further investigation of effects in adolescent girls.

Most of the studies investigating the effects of HIIT were conducted over 12 weeks, and none of them compared the effects of different program durations. Also, a large part of the data comes from studies conducted on people with excess body weight, while the number of studies on adolescent girls with normal body weight is still limited. Therefore, there is a need for additional studies that would examine how different durations of HIIT programs affect cardiometabolic parameters and aerobic capacity in this particular population. For this very reason, the implementation of interventions that last 6 and 8 weeks in this research represents an important contribution to the literature, as it allows insight into whether shorter protocols can lead to significant improvements in body composition and physical fitness. Given the importance of cardiometabolic health during adolescence, it is critical to investigate how various durations of high-intensity interval training [HIIT] affect key health indicators in adolescents. Therefore, the aim of this study was to investigate the effects of different durations of HIIT on cardiometabolic health and aerobic capacity in adolescent women.

## 2. Materials and Methods

### 2.1. Study Design

**Figure 1:**
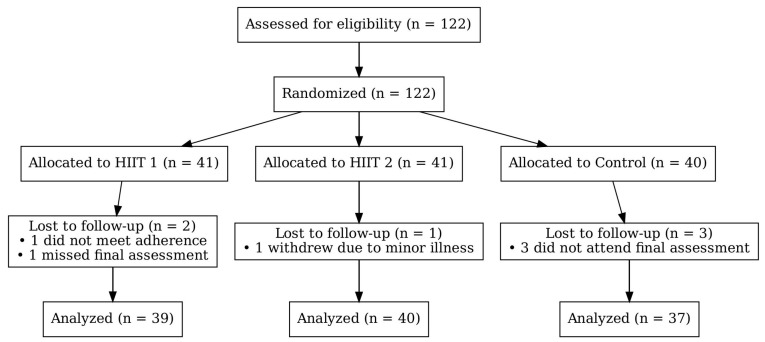
Experimental grouping CONSORT diagram.

The study was conducted as an experimental study with pre- and post-intervention measurements, which included three groups: two experimental and one control group. Using G*Power v3.1 software (Bonn, Germany, Bonn FRG, University of Bonn), the required sample size was estimated following the guidelines provided in scientific literature [19], based on the statistical analysis describing the scores of athletes in similar or equal variables during HIIT [15,16,17]. Consequently, for a type 1 error α = 0.05 and a power 1-β = 0.80, a sample size of 57 to 89 was considered necessary to detect significant effects. Therefore, sample of 122 participants represented a satisfactory sample. The flow of participants through the trial, including randomization, exclusions, dropouts, and final numbers analyzed, is shown in Figure 1 (CONSORT diagram). The aim of the intervention was to examine the effect of different durations of HIIT on body composition, lipid profile, blood pressure, and maximal oxygen consumption. Group HIIT 1 conducted a HIIT program for 6 weeks, while group HIIT 2 did the same program for 8 weeks. Both interventions were conducted twice a week.

Within the framework of the methodology of this study, the subjects were divided into three groups: experimental group 1 (HIIT 6 weeks), experimental group 2 (HIIT 8 weeks), and a control group that did not conduct additional training. There were no changes in the protocol during the study. Pre- and post-intervention assessments included body height, body mass, BMI, biochemical parameters (glucose, triglycerides, total cholesterol, HDL, and LDL), as well as a shuttle run test to assess VO_2max_.

### 2.2. Participants

A total of 122 healthy young women were enrolled, recruited via targeted social media outreach and personal referrals within the community. The following inclusion criteria were implemented: (1) age 17–19, as this age group was underrepresented in previous studies; (2) BMI between 18.5 and 24.9 kg/m^2^; and (3) physically active for at least 150 min each week. Exclusion criteria included: (1) exercise contraindications or restrictions; (2) acute illness or sports injuries; (3) psychiatric or other psychological disorders; (4) congenital or acquired heart disease; (5) a history of body mass loss procedures, surgical or hormonal; (6) use of any medications known to affect metabolism; and (7) absence from more than 10% of planned training sessions during the program.

Before the study began, we informed every participant about the training program’s risks and requirements, and we received signed informed permission from participants and their guardians.

A total of 122 subjects participated in the research, who after the initial testing were randomly assigned to three groups: experimental group 1 (HIIT 1 = 41), experimental group 2 (HIIT 2 = 41), and control group (C = 40). Following recruiting, eligible individuals (N = 122) were randomized to three groups using computer-generated randomization with SPSS software (version 26). A basic randomization approach with a 1:1:1 allocation ratio was used, with no stratification or blocking. An impartial researcher who was not involved in recruitment or assessments designed the randomization sequence to ensure concealment and prevent selection bias. Group assignments were revealed only after baseline measurements had been completed.

The Ethics Committee of the University agreed to the study (Approval No. 340/23, date of approval 2 February 2023) and registered it in the Open Science Framework (OSF) registry (registration DOI: https://doi.org/10.17605/OSF.IO/E7JAU). Prior to the trial, all participants supplied informed consent and filled out the International Physical Activity Questionnaire (IPAQ) [20,21]. There were no significant baseline differences between the experimental and control groups in terms of age, height, weight, BMI, or body fat percentage.

During the study, six participants dropped out (HIIT 1-1, HIIT 2-2, and C-3), and the final evaluation was completed by 117 participants (HIIT 1–39; HIIT 2–38; C-40) (Figure 1).

### 2.3. Testing Procedures

Testing was divided into two phases: initial measurements before the program and final assessments 6 and 8 weeks later. Participants were assessed for their baseline anthropometrics, cardiometabolic health, and aerobic capacity. Pre- and post-intervention assessments included body composition (body height, body mass, BMI, and body fat percentage), biochemical parameters (glucose, triglycerides, total cholesterol, HDL, and LDL), blood pressure (systolic and diastolic), as well as a shuttle run test to assess VO_2max_.

The training program lasted from April to June 2025, while every sample was examined in conformity with established protocols and standards of practice. Every measurement and the entire research project were carried out by the same investigators. Furthermore, all measurements were taken at the same time of day and according to the same test protocol. Although the same investigators conducted pre- and post-intervention measurements, they were blinded to group allocation. All testing followed standardized protocols, with participants instructed identically at each assessment and measurements performed under controlled conditions to minimize potential bias.

Prior to the testing methodology, all participants attended an introductory session where they were instructed on the proper protocol for each test. Because it is known that the type of warm-up influences performance [22], a standardized 15-min warm-up with dynamic exercises was performed before to testing. In the morning, body composition was first measured; then cardiometabolic health parameters were measured and, after a break, aerobic capacity, i.e., shuttle run test.

#### 2.3.1. Body Composition

As part of the anthropometric assessment, body mass (kg), body mass index (BMI, kg/m^2^), and body fat percentage (%) were measured. Measurements were conducted in accordance with the International Standards for Anthropometric Assessment (ISAK), using validated and standardized equipment. Body mass and body fat percentage were determined using the TANITA UM-72 analyzer, which uses the bioelectrical impedance (BIA) method. All measurements were performed in the morning, with previously provided standardized conditions, including the state of optimal hydration. Body mass index (BMI) was calculated according to the standard formula: BMI = body mass (kg)/height^2^ (m^2^). All equipment was calibrated before the beginning of the measurements, and assessments were performed by ISAK-certified anthropometrics, which ensured high reliability and accuracy of the obtained data.

#### 2.3.2. Cardiometabolic Health (GL, TG, SBP, DBP, TC, HDL, and LDL)

For the purposes of assessing lipid status, venous blood samples were collected in the morning, between 8:00 and 10:00 a.m., after a previous fasting period that lasted between 9 and 12 h. The collection and analysis of samples were carried out in cooperation with the Bosphorus Polyclinic in Novi Pazar, and the entire process was carried out by authorized medical laboratory experts. Samples were taken from the cubital vein in the sitting position of the subject, with the correct positioning of the hand for optimal access to the vein and facilitated performance of venipuncture.

Concentrations of total cholesterol and triglycerides were determined using a standard colorimetric method, which is based on spectrophotometric detection of colored complexes formed by enzymatic reactions. These methods have been routinely applied due to their reliability, reproducibility, and wide application in clinical biochemical analyses. On the other hand, the concentrations of high-density lipoprotein fractions (HDL) and low-density lipoprotein (LDL) were estimated by a direct enzymatic method, which allows greater analytical accuracy in the presence of variable biochemical properties of lipoprotein subfractions, which is particularly significant in populations with disturbed lipid homeostasis [23]. When applicable, LDL-C concentration was additionally calculated automatically via the analyzer’s integrated software system, using the validated formula: LDL (mmol/L) = total cholesterol − HDL (triglycerides/2.2) [24]. This formula, although limited at high triglyceride concentrations, still represents a standard and accepted method in clinical practice. The laboratory where the analyses were conducted was equipped with modern reagents for direct determination of LDL, which additionally ensured high reliability, validity, and precision of the obtained results. In addition to biochemical analyses, the measurement of arterial blood pressure and pulse frequency was an important part of the evaluation of the cardiometabolic status of the subjects. Systolic (SBP) and diastolic (DBP) blood pressure were determined using a validated automatic blood pressure measuring device (OMRON Healthcare Co., Ltd., Kyoto, Japan), which functions according to the principle of the oscillometer method. The validity and reliability of the instrument have been confirmed through research [25]. The measurements were performed under controlled conditions, after the subjects had spent at least 5 min at rest in a sitting position, in order to minimize external factors that could affect the results. For each participant, measurements were taken twice, 5 min apart, on the left upper arm, using appropriately sized cuffs adapted to the circumference of the upper arm, to ensure optimal measurement accuracy [23,26]. As a final result, the lower values from the two measurements of SBP and DBP were used, in accordance with recommendations from the literature indicating that the first-read result when using oscillometer devices can be elevated due to the initial tension and adaptation of blood vessels [27].

All biochemical analyses, both in the initial and final phases of the research, were conducted under uniform conditions, within 5 days before the start and after the end of the intervention program. The training intervention was conducted in the period from April to July 2025, and all samples were analyzed in accordance with valid laboratory protocols and standards of good practice.

#### 2.3.3. Aerobic Capacity (VO_2max_)

The shuttle run rest (SRT), also known as the “beep test” or the 20-min progressive run test, was used to assess aerobic capacity. This test involves continuously running back and forth between two parallel lines that are 20 m apart. During the test, participants run to the rhythm of a beep that determines the pace and progressively increases the intensity. The test is structured in several stages (levels), each of which lasts approximately 1 min and contains several runs (shuttles) 20 m long. In each subsequent stage, the speed is gradually increased, and the test ends when the subject fails to cover a distance of 20 m in the stipulated time interval in two consecutive attempts or ends the test arbitrarily due to exhaustion. The maximum number of levels successfully completed by the participant before the interruption is used as the test result. Based on that data, maximum oxygen consumption (VO_2max_) was estimated using the following equation: VO_2max_ = 31.025 + 3.238 × X − 3.248 × A + 0.1536 × A × X, where X indicates the speed in the last completed level (in km/h) and A represents the age of the subject in years. The necessary parameters for calculation are taken from standardized tables. The validity of this test for assessing cardiorespiratory endurance has been confirmed in previous research [28].

### 2.4. Experimental Program

The training protocols for both experimental groups were conducted twice a week (Table 1). The training began with a 10-min warm-up that included 5 min of easy jogging, 5 min of progressive acceleration, and 5 min of dynamic stretching. The main part of the training consisted of work intervals at an intensity of 90–95% of maximum heart rate (HR_max_), with active recovery phases at 50–55% of HR_max_. During the first 2 weeks, participants performed two sets of six intervals each, with a duration of 30 s of work and 30 s of recovery. In the third and fourth weeks, the number of intervals was increased to eight sets, while in the fifth and sixth weeks, they performed 10 intervals each. The rest period between sets was 4 min. The HIIT 1 group completed the protocol after the sixth week, while the HIIT 2 group continued with the same structure for an additional 2 weeks. Each training session ended with a 15-min cooldown phase, consisting of 10 min of easy running and 5 min of dynamic stretching. Throughout the program, a Polar 610i heart rate monitor (Finland) was used to ensure that participants consistently met the prescribed exercise intensity. During work intervals, participants in the HIIT1 group achieved an average heart rate of 185 ± 4 bpm, with a peak value of 192 ± 3 bpm, while those in the HIIT2 group reached an average of 186 ± 4 bpm and a peak of 193 ± 3 bpm. In phases of active recovery, heart rate remained at approximately 50–55% of HRmax (≈101–112 bpm). These values correspond to the planned intensity zone of 90–95% HRmax for this age group and confirm that both groups consistently adhered to the prescribed training load. Except for one participant in the HIIT1 group, all individuals completed ≥95% of the scheduled training sessions, with sessions following the intended work-to-rest intervals and structure, thereby demonstrating the high fidelity of the intervention.

### 2.5. Statistical Analysis

All data have been described using mean, standard deviation, and 95% confidence interval. Using Kolmogorov–Smirnov test, all variables have been subjected to the normality of distribution. Two variables showed mild deviations from normality, but due to the robustness of ANOVA on normality assumption, parametric methods (ANOVA) have been chosen for further statistical analysis. The assumption of sphericity was checked, and when violated, the Greenhouse–Geisser correction was applied. Considering the experimental design, two-way 3 × 2 between-within ANOVA was applied to identify effects of between-groups factor Group (two experimental and control group) and within-groups factor Time (initial and final measurement). Between subject’s ANOVA was applied to identify significance in initial baseline differences. Bonferroni post hoc correction, together with additional Cohen’s d if particular difference appeared to be significant, was applied to estimate significance of particular differences. Using Delphi approach, minimal clinically important differences (MCID) have been estimated for BMI and blood pressure variables. Partial eta squared has been used as an effect size measure, and it is interpreted as small if less than 0.06, moderate if greater or equal to 0.06 and smaller than 0.15, and large if greater or equal to 0.15. Type I error was set at α = 5%. All data has been calculated using Statistica 14 (Cloud Software Group, Inc.—Palo Alto, CA, USA (2023), Data Science Workbench).

## 3. Results

Between-subjects ANOVA revealed no significant initial baseline differences in all observed variables with *p*-values ranging from 0.47 to 0.98, pointing to the non-existence of baseline measurements covariate effect. Observing Table 2 and Table 3, it can be seen that both the HIIT 1 and HIIT 2 programs of HIIT led to statistically significant improvements in most monitored parameters compared to the control group. A decrease in body weight, BMI, and body fat percentage (all *p* < 0.001), as well as improvements in total cholesterol, triglycerides, LDL cholesterol, and systolic and diastolic blood pressure (all *p* < 0.001), were recorded. Changes in glycemia and HDL cholesterol were not statistically significant. Also, a significant increase in aerobic capacity, expressed through VO_2max_ (*p* < 0.001), was recorded. A detailed presentation of all results is shown in Table 1 and Table 4.

Main effect of factor Measurement appeared to be significant on variables BM, BMI, and BF with large effect size. Similarly, the main effect of factor Group was found to be significant for variables BM; BMI and BF also have a large effect size. Bonferroni post hoc correction revealed that the control group differs from both experimental groups.

## 4. Discussion

The aim of this study was to examine the effects of different durations of HIIT on cardiometabolic health and aerobic capacity in adolescent women. Given the increasing need for effective methods of physical activity in adolescents, the results of this study provide a significant contribution to understanding the effects of HIIT program duration on body composition, cardiometabolic health, and aerobic capacity in young women. These results are particularly relevant given that HIIT programs are highly adaptable and can be easily integrated into school and club settings, making them a practical solution for improving the health and physical fitness of children and adolescents [29,30,31,32,33,34,35,36]. In addition to significantly improving cardiorespiratory fitness, strength, agility, and body composition, HIIT also helps reduce the risk of obesity and chronic diseases among youth [30,33,34,35,36,37,38]. It is particularly important that these programs are well received by both students and teachers, with motivation and enjoyment increasing when students are involved in designing the exercises or when diverse content and music are incorporated [30,32,37]. HIIT can be adapted to different conditions—from running and circuit training to games and strength exercises—allowing teachers and coaches to implement regardless of space or equipment availability [30,32]. Studies show that such programs are particularly beneficial for girls and less active students, who may need additional support to reach the required intensity and remain motivated [30,32,37]. Introducing HIIT into schools and sports clubs represents an effective, accessible, and motivating approach to improve youth health and prevent modern health problems. The findings indicate that both 6-week and 8-week HIIT programs lead to statistically significant improvements in most of the parameters examined, including reductions in body mass (*p* < 0.001), BMI (*p* < 0.001), and body fat percentage (*p* < 0.001), as well as improvements in the lipid profile (total cholesterol, *p* < 0.001) and triglycerides (*p* < 0.001), blood pressure—systolic (*p* < 0.001) and diastolic (*p* < 0.001), as well as maximal oxygen consumption—VO_2max_ (*p* < 0.001). Statistically significant changes were not recorded in glucose levels (*p* = 0.078) and HDL cholesterol (*p* = 0.825). It should be noted that the MCID thresholds used in this study (1 kg/m^2^ for BMI, 7.5 mmHg for systolic blood pressure, and 4.5 mmHg for diastolic blood pressure) represent expert-based approximations derived through the Delphi approach and serve primarily as reference values for clinical interpretation rather than absolute cutoff points. In this context, the reduction of BMI by 1.1 kg/m^2^ in the HIIT groups exceeded the proposed threshold of 1 kg/m^2^, suggesting that the observed change can be considered clinically meaningful. Similarly, reductions in systolic and diastolic blood pressure also surpassed their respective MCID values, further confirming the clinical relevance of the intervention. These findings indicate that the observed improvements were not only statistically significant but also potentially of clinical importance. The lack of significant changes in HDL and glycemia does not diminish their clinical relevance, as both are well-established indicators of elevated cardiometabolic risk in adolescents [39,40,41,42,43,44]. The absence of changes may be explained by the short duration of the intervention, normal baseline values, and physiological fluctuations during adolescence [39,43,45,46]. Nevertheless, persistently low HDL and elevated glycemia, as well as combined indices such as the TG/HDL-C ratio, remain key predictors of long-term cardiometabolic risk [40,41,47,48]. These improvements confirm previous research on the effectiveness of HIIT as a time-efficient method for improving health indicators in young people, but at the same time offer new insight into the potential of shorter protocols, which may be practical for implementation in the adolescent population.

These findings are consistent with previous studies confirming the beneficial effects of high-intensity interval training (HIIT) on metabolic and cardiovascular health in adolescents. For example, several authors have reported similar benefits of HIIT programs in young adults, further supporting the results of our studies [49,50,51,52]. Guo, Chen, and Yuan [53] showed that after just 4 weeks of HIIT, there was a significant reduction in waist circumference and body fat percentage in overweight young women, with the first changes being observed after just one week. Our results are consistent with these findings, confirming that even shorter HIIT interventions, including a 6-week protocol, can lead to rapid and clinically relevant improvements in body composition. Similarly, Sun et al. [54] found significant reductions in body mass (*p* = 0.021; ES = 0.19), percentage of body fat (*p* = 0.037; ES = 0.17), and visceral fat (*p* = 0.005 with ES), using a HIIT protocol (three times per week, 85–95% HR_max_), which was reduced from 0.005, total body fat percentage and BMI. It is important to emphasize that the effects in our study were achieved without dietary control, which is consistent with the results of Khammassi et al. [55], who showed that physical activity alone, without a calorie restriction regimen, can lead to significant improvements in body composition over a 12-week HIIT program. However, there is research that suggests that shorter interventions may not always result in significant effects. For example, Baquet et al. [56] conducted a 10-week HIIT program in adolescents but did not find statistically significant changes in body fat percentage compared to a control group. These results indicate that the effects of HIIT may depend on the frequency and intensity of the load, as well as on individual characteristics of the subjects. In comparing the two experimental groups (HIIT 1 and HIIT 2), significant differences were observed only in the values of triglycerides (TG) and total cholesterol (TC), with the 8-week program (HIIT 2) demonstrating a more pronounced effect. This outcome can be explained by the cumulative impact of high-intensity intervals on lipid metabolism, as adaptation of the lipoprotein system requires a longer period of continuous stimulation. Our findings confirm that reductions in TG and TC levels require additional stimuli and longer program durations, which is consistent with previous research indicating that interventions lasting 8 weeks or more produce more pronounced effects on lipid status [51,55]. Additionally, Frimpong et al. [16] demonstrated that even low-volume but frequent HIIT protocols can lead to favorable metabolic changes in women with elevated BMI. Our results are particularly in line with the findings of Khammassi et al. [55], who reported average reductions of 27% for triglycerides, 12.4% for total cholesterol, and 12% for LDL-C. On the other hand, changes in HDL cholesterol were not statistically significant, which also corresponds with our findings. In contrast, Racil et al. [51] observed a significant increase in HDL-C (+6.3%) and a reduction in insulin resistance in obese adolescent girls following a 12-week HIIT program, suggesting potential additional benefits when specific population characteristics are present or when exercise is combined with complementary components such as dietary counseling. Overall, it can be concluded that lipid parameters require extended and consistent intervention, while VO_2max_ and body composition tend to respond more rapidly to shorter HIIT protocols. Our results are also consistent with the findings of Sun et al. [54], who reported significant reductions in systolic (*p* = 0.018; ES = 0.84) and diastolic blood pressure (*p* = 0.008; ES = 1.76), as well as triglycerides (*p* = 0.004; ES = 1.33) following a HIIT cardio intervention, confirming the effectiveness of this type of training. Both the 6-week and 8-week HIIT interventions resulting in statistically significant improvements in aerobic capacity were recorded, with no significant difference between the groups, suggesting that adaptations in this parameter can occur in the early stages of the intervention. The 6- to 8-week period is sufficient because adolescents respond quickly to HIIT, showing early improvements in VO_2max_, body composition, and metabolic markers due to rapid cardiovascular, muscular, and metabolic adaptations [12,57,58,59,60]. Longer programs may enhance these effects, but most key gains occur within this time frame [12,36,59,60]. Early neuromuscular and cardiovascular adjustments are usually observed after 6–8 weeks of consistent training. Lundby and Jacobs [61] highlight that these early changes are driven by increased mitochondrial enzyme activity, improved capillary perfusion, and more efficient metabolism. Adolescents respond more quickly to such programs due to greater physiological plasticity, enabling early improvements in body composition, lipid profile, blood pressure regulation, and aerobic capacity. However, a longer training duration is required for more sustained structural and metabolic adaptations. While HIIT proved effective over 6–8 weeks, the absence of long-term follow-up remains a limitation, as it prevents firm conclusions about the persistence of these benefits beyond the intervention period [58]. Our results align with previous studies that have confirmed the effectiveness of HIIT in improving cardiorespiratory fitness. During the 8-week intervention, an approximate increase of 18% in VO_2max_ was observed [15], while Lu et al. [62] demonstrated that a 12-week HIIT-R protocol increased VO_2max_ by 17.1% in untrained female students. Similarly, Jovanović et al. [4] reported a significant improvement in shuttle run test results (*p* < 0.01; ηp^2^ = 0.111) following a 12-week HIIT program in adolescents. These findings confirm that cardiovascular adaptations can be achieved relatively quickly through high-intensity interval training. Additionally, our results are consistent with those of Guo, Chen, and Yuan [53], who showed that even 4 weeks of HIIT can significantly improve VO_2max_ in young women with overweight. Likewise, Kong et al. [63] documented rapid improvements in cardiovascular function after 5 weeks of Wingate-based HIIT, although without statistical significance, while Racil et al. [51] reported a statistically significant increase in VO_2max_ (*p* = 0.019; ES = 0.48) following a 12-week program. Our findings contribute to this body of evidence, emphasizing that even shorter HIIT protocols (6 and 8 weeks) can significantly improve aerobic capacity in adolescent girls. Finally, the results of Plavšić et al. [64] suggest that the combination of HIIT and dietary interventions may lead to additional improvements in cardiometabolic markers, including insulin resistance (HOMA-IR), hsCRP, and AUC parameter, indicating the potential for even more pronounced effects when HIIT is combined with other intervention modalities.

The results of this study indicate that different durations of HIIT programs (6 and 8 weeks) can have a significant positive impact on body composition, cardiometabolic health, and aerobic capacity in adolescent girls. However, a limitation of this study is that VO_2max_ was estimated using the shuttle run test, an indirect method that can be influenced by motivation and testing conditions, particularly in adolescents. Our findings demonstrate that shorter HIIT protocols are sufficient to improve VO_2max_ and reduce body mass, while longer protocols are more effective in improving lipid profiles, particularly triglycerides and total cholesterol. These insights have practical value for coaches, instructors, healthcare professionals, and others working with physically active adolescent females, as they enable effective planning of training programs based on specific goals whether the focus is on fat reduction, endurance enhancement, or prevention of cardiometabolic disorders. Moreover, the results can serve as a foundation for designing individualized training interventions both for active girls and those at risk of inactivity and metabolic imbalance. This study also provides a basis for future research to explore the optimal duration, frequency, and structure of HIIT programs in adolescent female populations, as well as the potential benefits of combining HIIT with other components, such as nutritional guidance or resistance training. Future studies are encouraged to examine the long-term effects of HIIT, along with psychological factors such as motivation and adherence to training protocols among young females. While HIIT proved effective over 6–8 weeks, the absence of long-term follow-up remains a limitation, as it prevents firm conclusions about the persistence of these benefits beyond the intervention period [58].

## 5. Conclusions

The results of this study confirm that high-intensity interval training implemented over a period of 6 to 8 weeks significantly contributes to improving cardiometabolic health and aerobic capacity in adolescent girls aged 17 to 19. Both HIIT protocols led to statistically significant reductions in body mass, BMI, and body fat percentage, as well as improvements in total cholesterol, triglycerides, LDL cholesterol, and systolic and diastolic blood pressure. Increases in VO_2max_ were confirmed in both experimental groups, while changes in glycemia and HDL cholesterol were not statistically significant. Notably, the 8-week program demonstrated a stronger effect in reducing total cholesterol and triglyceride levels compared to the 6-week program [HIIT 1], highlighting the importance of intervention duration when targeting improvements in lipid profiles. These findings suggest that parameters such as VO_2max_ and body composition can be improved in a shorter period, while deeper metabolic changes require longer and more consistent training. In conclusion, HIIT proved to be a time-efficient, accessible, and practical strategy for improving the health of young women, even in the absence of dietary control or specialized equipment. These results may serve as a foundation for further research and the implementation of HIIT programs in the adolescent population. We acknowledge that our study has limitations that should be addressed when interpreting the findings. The study population consisted mainly of young women aged 17 to 19 with a normal BMI. While this enhances internal validity, it restricts the findings’ generalizability to other populations, such as overweight, sedentary, or elderly people. In addition, potential confounding variables, like dietary habits, sleep quality, menstrual cycle phase, physical activity, and socioeconomic level, were not controlled or accounted for in this study. These factors may have influenced cardiometabolic results and should be explored in future studies.

## Figures and Tables

**Table 1:** Basic descriptive characteristics in the control and experimental groups and in the initial and final measurements.

Outcome	Initial		
	HIIT 1	HIIT 2	C
Age	18.65 ± 0.87	18.99 ± 0.74	18.74 ± 0.42
BH [cm]	173.42 ± 9.91	174.15 ± 8.47	174.33 ± 9.54
BM [kg]	78.49 ± 2.23	78.71 ± 2.26	78.52 ± 2.24

**Table 2:** Changes in body composition, cardiometabolic health parameters, and aerobic capacity between initial and final measurements across the HIIT 1, HIIT 2, and control groups.

	Initial Measurement			Final Measurement		
	HIIT 1	HIIT 2	C	HIIT 1	HIIT 2	C
	M ± SD 95% CI	M ± SD 95% CI	M ± SD 95% CI	M ± SD 95% CI	M ± SD 95% CI	M ± SD 95% CI
BM	78.49 ± 2.23 [77.80, 79.18]	78.71 ± 2.26 [78.01, 79.41]	78.52 ± 2.24 [77.83, 79.21]	74.73 ± 1.95 [74.13, 75.33]	73.88 ± 2.44 [73.12, 74.64]	78.56 ± 2.25 [77.86, 79.26]
BMI	24.02 ± 1.06 [23.69, 24.35]	23.64 ± 0.99 [23.33, 23.95]	24.49 ± 1.02 [24.17, 24.81]	23.11 ± 1.32 [22.70, 23.52]	22.96 ± 1.56 [22.48, 23.44]	24.13 ± 1.57 [23.64, 24.62]
BF	23.03 ± 0.79 [22.78, 23.28]	23.14 ± 0.90 [22.86, 23.42]	23.04 ± 0.79 [22.79, 23.29]	20.96 ± 0.59 [20.78, 21.14]	20.73 ± 0.87 [20.46, 21.00]	23.10 ± 0.77 [22.86, 23.34]
GL	4.69 ± 0.29 [4.60, 4.78]	4.88 ± 0.41 [4.75, 5.01]	4.71 ± 0.31 [4.61, 4.81]	4.49 ± 0.25 [4.41, 4.57]	4.33 ± 0.38 [4.21, 4.45]	4.75 ± 0.32 [4.65, 4.85]
TG	125.13 ± 1.68 [124.61, 125.65]	125.25 ± 1.50 [124.78, 125.72]	125.14 ± 1.69 [124.62, 125.66]	88.09 ± 4.63 [86.66, 89.52]	85.25 ± 0.94 [84.96, 85.54]	125.15 ± 1.71 [124.62, 125.68]
SBP	129.10 ± 1.84 [128.53, 129.67]	129.34 ± 1.70 [128.81, 129.87]	129.15 ± 1.86 [128.57, 129.73]	121.59 ± 2.85 [120.71, 122.47]	119.84 ± 1.87 [119.26, 120.42]	129.59 ± 2.09 [128.94, 130.24]
DBP	78.26 ± 1.60 [77.76, 78.76]	78.18 ± 1.57 [77.69, 78.67]	77.43 ± 3.73 [76.27, 78.59]	66.00 ± 3.11 [65.04, 66.96]	64.82 ± 1.20 [64.45, 65.19]	78.25 ± 1.69 [77.73, 78.77]
TC	170.00 ± 1.53 [169.53, 170.47]	169.88 ± 1.54 [169.40, 170.36]	170.02 ± 1.54 [169.54, 170.50]	148.31 ± 2.80 [147.44, 149.18]	145.70 ± 3.48 [144.62, 146.78]	169.51 ± 3.86 [168.31, 170.71]
HDL	34.66 ± 0.77 [34.42, 34.90]	34.59 ± 0.63 [34.39, 34.79]	34.66 ± 0.77 [34.42, 34.90]	34.82 ± 0.60 [34.63, 35.01]	35.05 ± 0.80 [34.80, 35.30]	34.83 ± 0.60 [34.64, 35.02]
LDL	112.26 ± 1.33 [111.85, 112.67]	112.18 ± 1.35 [111.76, 112.60]	113.00 ± 3.06 [112.05, 113.95]	96.04 ± 1.17 [95.68, 96.40]	94.92 ± 2.02 [94.29, 95.55]	112.52 ± 3.51 [111.43, 113.61]
VO_2max_	39.45 ± 3.44 [38.38, 40.52]	39.55 ± 3.32 [38.52, 40.58]	39.73 ± 3.40 [38.68, 40.78]	45.88 ± 2.53 [45.10, 46.66]	46.74 ± 3.26 [45.73, 47.75]	39.78 ± 3.26 [38.77, 40.79]

Legend: M ± SD—mean ± standard deviation; 95% CI—95% confidence interval, BM—body mass; BMI—body mass index; BF—body fat percentage; GL—glucose; TG—triglycerides; SBP—systolic blood pressure; DBP—diastolic blood pressure; TC—total cholesterol; HDL—high-density lipoprotein; LDL—low-density lipoprotein; VO_2_max—maximal oxygen.

**Table 3:** Two-way mixed ANOVA results, Bonferroni post hoc correction, together with additional Cohen’s d effect size for significant particular differences (Con vs. HIIT 1, Con vs. HIIT 2, and HIIT 1 vs. HIIT 2) for the effects of Group and Time (initial vs. final) on body composition, cardiometabolic health parameters, and aerobic capacity.

	Measurement Effect			Group Effect			
	F	*p*	η^2^	F	*p*	η^2^	Bonferroni Post Hoc Cohen’s d
BM	374.814	<0.001	0.599	12.418	<0.001	0.153	0.372; 0.426; 0.229
BMI	262.113	<0.001	0.691	17.235	<0.001	0.233	0.395; 0.471
BF ^¥^	497.225	<0.001	0.816	30.622	<0.001	0.351	0.197; 0.263
GL	96.179	<0.001	0.455	2.612	0.078	0.043	None
TG	6916.821	<0.001	0.894	1755.365	<0.001	0.968	0.671; 0.791; 0.421
SBP ^¥^	742.319	<0.001	0.868	83.709	<0.001	0.593	0.242; 0.199
DBP ^¥^	865.912	<0.001	0.885	141.735	<0.001	0.700	0.138; 0.244
TC	2001.049	<0.001	0.946	491.477	<0.001	0.895	0.411; 0.492; 0.221
HDL	32.091	<0.001	0.218	0.193	0.825	0.003	None
LDL	1668.500	<0.001	0.936	363.755	<0.001	0.864	0.265; 0.334
VO_2max_	138.564	<0.001	0.546	22.577	<0.001	0.282	0.183; 0.544

Legend: *p*—*p*-value; η^2^—partial eta squared; BM—body mass; BMI—body mass index; BF—body fat percentage; GL—glucose; TG—triglycerides; SBP—systolic blood pressure; DBP—diastolic blood pressure; TC—total cholesterol; HDL—high-density lipoprotein; LDL—low-density lipoprotein; VO_2max_—maximal oxygen; ^¥^—Greenhouse–Geisser degrees of freedom correction applied.

**Table 4:** Summary of high-intensity interval training programs for both groups.

	HIIT 1 (6 Weeks) Intensity/Recovery	HIIT 2 (8 Weeks) Intensity/Recovery
Warm-up	5 min of jogging 5 min of acceleration running 5 min of dynamic stretching	5 min of jogging 5 min of acceleration running 5 min of dynamic stretching
Main stimuli	90–95%/50–55% HR_max_ Week 1–2: 2 sets × 6 (30/30 s) Week 3–4: 2 sets × 7 (30/30 s) Week 5–6: 2 sets × 8 (30/30 s) Passive recovery between sets: 4 min	90–95%/50–55% HR_max_ Week 1–2: 2 sets × 6 (30/30 s) Week 3–4: 2 sets × 7 (30/30 s) Week 5–6: 2 sets × 8 (30/30 s) Week 7–8: 2 sets × 9 (30/30 s) Passive recovery between sets: 4 min
Cooldown	10 min of running 5 min of dynamic stretching exercises	

## Data Availability

Data are contained within the article.
